# Association between blood pressure control in hypertension and urine sodium to potassium ratio: From the Korea National Health and Nutrition Examination Survey (2016–2021)

**DOI:** 10.1371/journal.pone.0314531

**Published:** 2024-11-26

**Authors:** Youngmin Yoon, Minkook Son

**Affiliations:** 1 Division of Nephrology, Department of Medicine, Chosun university hospital, Chosun University School of Medicine, Gwang-ju, Korea; 2 Department of Physiology, Dong-A University College of Medicine, Busan, Korea; 3 Department of Data Sciences Convergence, Dong-A University Interdisciplinary Program, Busan, Korea; New York University Grossman School of Medicine, UNITED STATES OF AMERICA

## Abstract

**Background:**

Hypertension (HTN) is linked to an enhanced risk of chronic kidney disease and cardiovascular disease. While sodium and potassium intake affect blood pressure (BP) control, the urine sodium-to-potassium (Na/K) ratio, which reflects dietary balance and renal regulation of these electrolytes, could be associated with BP. This study aimed to evaluate the independent association between urine Na/K and uncontrolled HTN.

**Methods:**

Data were collected from the Korea National Health and Nutrition Examination Survey from 2016 to 2021. A total of 5,770 participants diagnosed with HTN were enrolled in this study. Uncontrolled HTN was characterized by a systolic blood pressure (SBP) ≥ 140 mmHg or diastolic blood pressure (DBP) ≥ 90 mmHg. Logistic regression analysis was used to assess the relationship between urine Na/K and the risk of uncontrolled HTN.

**Results:**

The urine Na/K was positively correlated with both SBP and mean arterial pressure. Higher urine Na/K was significantly associated with an increased risk of uncontrolled HTN using both continuous (odds ratio [95% confidence interval] 1.13 [1.09–1.16], P <0.01]) and across quartile values (with Q1 as a reference; Q2: 1.26 [1.06–1.49], P = 0.01; Q3: 1.50 [1.27–1.78], P <0.01; Q4: 1.85 [1.55–2.17], P < 0.01). The subgroup analysis also showed that higher urine Na/K were significantly related to the risk of uncontrolled HTN in the presence of proteinuria or CKD.

**Conclusion:**

Urine Na/K ratio is independently associated with uncontrolled HTN in the general population and in patients with CKD. Our findings suggest that monitoring the urine Na/K could serve as an effective tool for identifying subjects at risk of uncontrolled HTN.

## Introduction

Hypertension (HTN) is a significant global health concern affecting an estimated 1.13 billion individuals worldwide [1]. In North America, the prevalence of HTN remains alarmingly high, affecting an estimated 49.64% of adults from 2017–2018 [2]. HTN is widely recognized as a major contributing factor to the development of various diseases, such as chronic kidney disease (CKD), diabetes, and cardiovascular disease (CVD), including stroke and coronary artery disease [3, 4]. Intensive blood pressure (BP) control is crucial for ameliorating the complications associated with HTN and improving patient outcomes, such as preserving renal function and reducing cardiovascular mortality [5–7]. The target BP for HTN suggested by the guidelines varies depending on cardiovascular risk, CKD, and age [8–10]. The American Heart Association recommends a target BP of less than 130/80 mmHg for most patients [8]. In contrast, the European Society of Cardiology guidelines recommend a target BP range of 120–129/80 mmHg for patients aged <65 years and 130–139/80 mmHg for those aged ≥ 65 years [9]. The Kidney Disease Improving Global Outcomes guidelines recommend a target systolic BP (SBP) of < 120 mmHg for most adults with HTN and CKD [10]. Despite these guidelines, the control rate remains low, with only 39.64% of Americans achieving well-controlled BP (mean SBP of < 130 mmHg and diastolic BP (DBP) of < 80 mmHg) [2]. Furthermore, on a global scale, less than half of individuals treated for HTN achieve adequate BP control, resulting in control rates of 23% for females and 18% for males (< 140/90 mmHg) [11]. Accurate BP measurement is crucial for effective management However, discrepancies between office BP and home BP measurements pose a significant challenge [12]. Previous studies have validated that the significant role of dietary and nutritional strategies in the prevention and management of HTN [13–15]. These strategies emphasize a dietary pattern rich in fiber, fruits, vegetables, whole grains, lean protein sources, and nuts while also stressing the importance of reducing sodium and saturated fats [16–18]. Notably, a diet high in salt is correlated with an elevated BP [19, 20], whereas a diet rich in potassium is associated with a lower BP [21, 22]. Consistent with the appropriate antihypertensive medications, diet plays a crucial role in the management of HTN. Nonetheless, current methods that rely on self-reported dietary intake may lack the precision required to assess an individual’s diet. The urinary sodium to potassium (Na/K) ratio is a valuable indicator that not only reflects the dietary balance of these electrolytes but could also be more closely associated with BP control compared to evaluating sodium or potassium intake individually. In this study, we aimed to evaluate the independent association between urine Na/K and uncontrolled HTN in the Korean population.

## Method

### Study population

This study was based on the Korea National Health and Nutrition Examination Survey (KNHANES), a nationwide, cross-sectional survey performed by the Korean Ministry of Health and Welfare. KNHANES data are freely available from Korea Centers for Disease Control and Prevention (https://knhanes.cdc.go.kr), and the data were accessed on March 4, 2024, for this study. The KNHANES collects comprehensive information on the health status, dietary intake, and health-related behaviors of the non-institutionalized Korean population. All participants provided informed consent willingly for their voluntary involvement, and the KNHANES received approval from the Institutional Review Board of Korea Centers for Disease Control and Prevention. The study’s protocol received approval from the Institutional Review Board of Chosun University Hospital in compliance with the Declaration of Helsinki (Approval Number: CHOSUN 2023-12-022).

**Fig 1:**
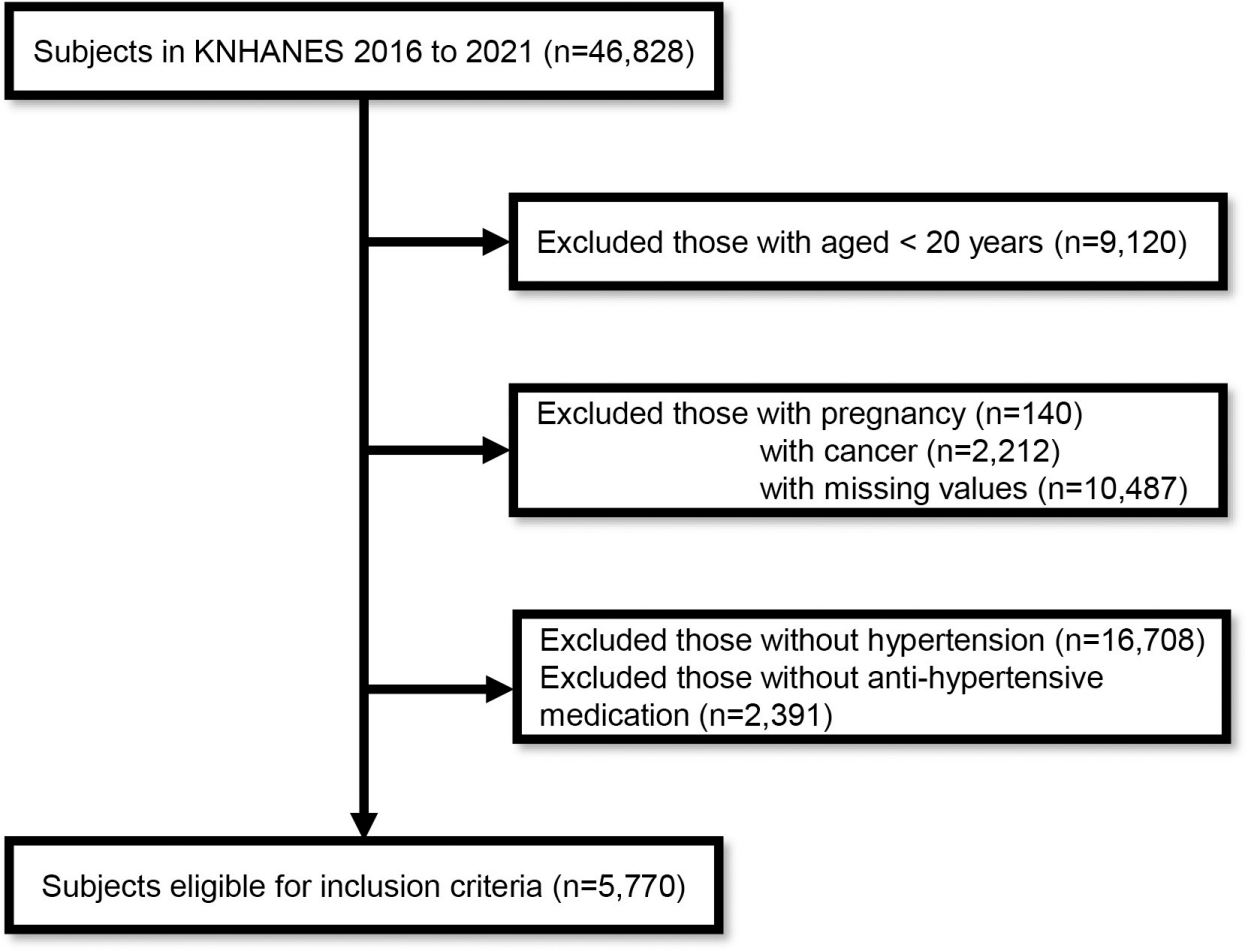
Flow diagram of study population.

For this analysis, we included 46,828 adults aged ≥20 years who participated in the KNHANES between 2016 and 2021. Subjects were excluded from this study if they were aged <20 years (n = 9,120), pregnant (n = 140), diagnosed with cancer (n = 2,212), had missing data (n = 10,487), were not previously diagnosed with HTN (n = 16,708), or were not taking antihypertensive medications (n = 2,391). Finally, A total of 5,770 participants were enrolled in the study (Fig 1).

### Medical history and demographic data

Demographic data, medical histories, and lifestyle factors were collected using self-report questionnaires and personal interviews conducted by trained research staff. For smoking status, participants were grouped into current smokers and nonsmokers, with the latter encompassing both former smokers and those who had never smoked. For alcohol consumption, participants were classified as non-alcohol drinkers if they had not consumed alcohol in the previous year or if they drank alcohol less frequently than once a month. Those who consumed alcohol more than once a month were considered alcohol drinkers. For exercise pattern, regular exercise was defined as meeting or exceeding, including either ≥ 150 min of moderately intense exercise per week or ≥ 75 min of vigorous exercise per week, or equivalent mixed exercise. Nonregular exercise encompassed activity levels below these thresholds. In this study, spot urine samples were collected from the subjects, and the urine Na and K levels were analyzed using the Labospect 008AS (Hitachi, Japan). proteinuria was diagnosed when a urine dipstick test detected protein levels higher than 1+.

### Blood pressure measurement and definition of uncontrolled hypertension

**Fig 2:**
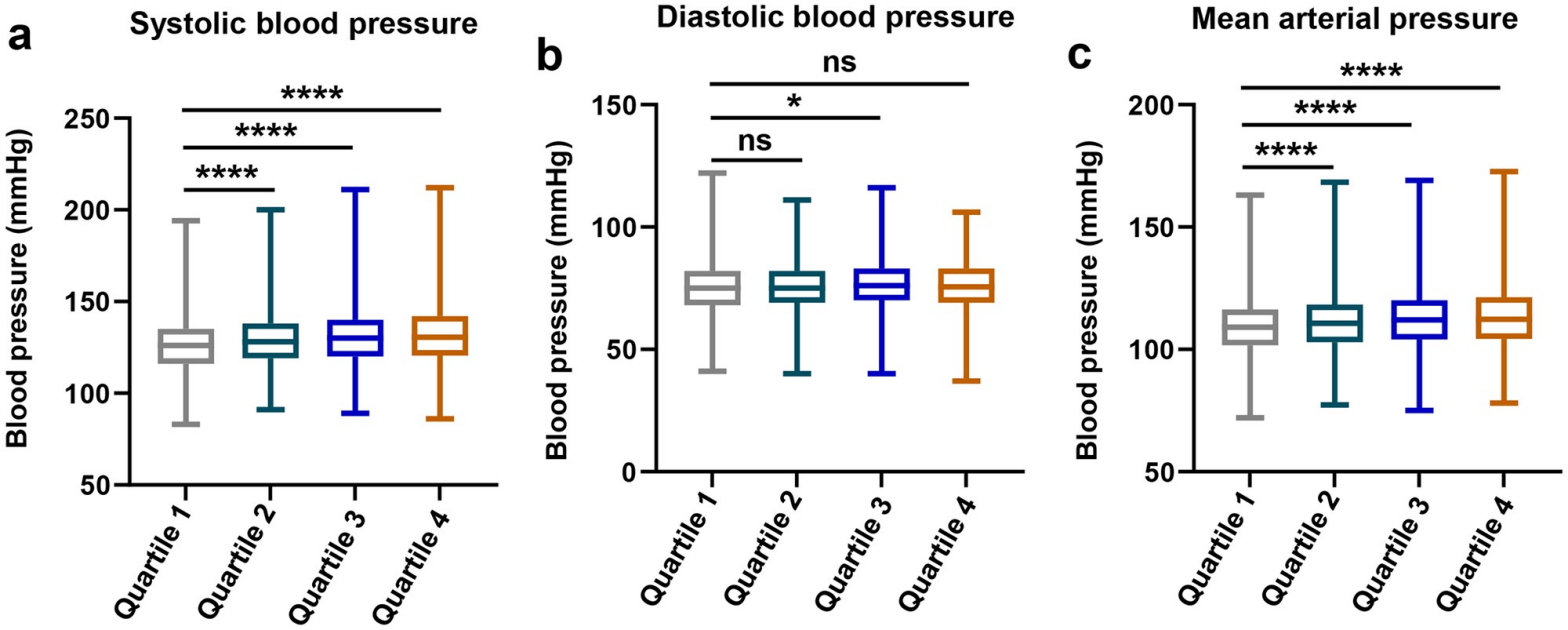
Boxplots of blood pressure across urine Na/K quartiles. The boxplots representing the distribution of systolic blood pressure (SBP), diastolic blood pressure (DBP) and mean arterial pressure (MAP) across different quartiles of the urine Na/K ratio. Each boxplot illustrates the median (central line), the interquartile range (box edges), and the range (whiskers), for each quartile. P values were calculated using one-way ANOVA with Tukey’s post-hoc analysis; *p< 0.05, ****p< 0.0001 and ns = not significant.

In the KNHANES, BP was measured by four trained nurses. Measurements were taken after participants had rested for 5 minutes in a sitting position with their feet flat on the floor and their arms supported at heart level [23]. From 2007 to 2019, a standard mercury sphygmomanometer (Baumanometer, WA Baum Co.) was used. In 2020, a mercury-free sphygmomanometer (Greenlight300) was implemented, and since 2021, an oscillometric device (Microlife WatchBP Office) has been used. Three BP measurements were taken at 30-second intervals, with the mean of the second and third measurements used for analysis [23]. The mean arterial pressure (MAP) was estimated by taking two-thirds of the DBP and adding one-third of the SBP. Given that the prevalence of isolated systolic hypertension increases with age [24], we conducted analyses using MAP in association with urine Na/K ratio, sodium intake, and potassium intake (Fig 2, S2 and S3 Figs).

We defined uncontrolled HTN as having a SBP of ≥ 140 mmHg or DBP of ≥ 90 mmHg in participants who were on antihypertensive medication [25]. Participants who reported taking antihypertensive medications daily were classified as adherent, whereas those who reported not taking medications daily were classified as poorly adherent.

### Definition of metabolic syndrome, proteinuria and CKD

Metabolic syndrome (MetS) was diagnosed when three or more of the following criteria were met [26, 27]: 1) abdominal obesity, defined as a waist circumference (WC) ≥ 90 cm for men or ≥ 85 cm for women, 2) triglycerides ≥ 150 mg/dL, or receiving medication for elevated triglycerides, 3) high-density lipoprotein (HDL)—cholesterol < 40 mg/dL for men or < 50 mg/dL for women, or undergoing treatment for reduced HDL cholesterol, 4) high BP, with a SBP ≥ 130 mmHg, or a DBP ≥ 85 mm Hg, or receiving antihypertensive therapy, 5) elevated fasting glucose ≥ 100 mg/dL, or drug treatment for hyperglycemia. Proteinuria was evaluated using a single dipstick urine test and was defined as having more than 1 positive result. The Modification of Diet in Renal Disease (MDRD) Study equation was used to calculate the estimated glomerular filtration rate (eGFR) [28]. CKD was defined as an eGFR of <60 mL/min/1.73 m^2^ [29]. CKD stage categorized based on eGFR values as follows: G3, 30–59 mL/min/1.73 m^2^; G4 and 5, eGFR < 30 mL/min/1.73 m^2^).

### Statistical analysis

Continuous variables were presented as the mean value along with the standard deviation, while categorical variables were described using the count of cases and their corresponding percentages. We used Student’s t-test or one-way analysis of variance (ANOVA) for continuous variables and chi-square tests for categorical variables to compare the baseline characteristics between the control and uncontrolled groups. To investigate the association between urine Na/K and BP control, we performed logistic regression analyses, presenting the results as odds ratios (OR) with 95% confidence intervals (CI). The multivariate analysis consisted of two adjusted models to account for potential confounding factors. Model 1 adjusted for variables such as sex, age, body mass index (BMI), waist circumference (WC), poor medication adherence, smoking status, alcohol consumption, regular exercise, diabetes, MetS, CVD, dyslipidemia, CKD, and sodium and potassium intake. Model 2 incorporated adjustments for sex, age, BMI, WC, poor medication adherence, smoking status, alcohol consumption, regular exercise, fasting glucose, total cholesterol, HDL-cholesterol, triglycerides, eGFR, and sodium and potassium intake. Model 1 was designed to include categorical variables such as CKD, DM, and dyslipidemia, while Model 2 incorporated continuous variables like fasting glucose and eGFR. Using these two models allowed us to explore the association between urine Na/K and uncontrolled HTN under different sets of adjustments, ensuring the robustness of our findings. Additionally, this approach enabled us to capture both the discrete and continuous aspects of related health conditions. All statistical analyses were performed using the SPSS software version 20 (IBM Corporation, Armonk, NY, USA) and Prism 10.1.2 (GraphPad). Graphs were generated using Prism 10.1.2 (GraphPad) and R 4.3.0 (https://www.r-project.org, R Foundation for Statistical Computing, Vienna, Austria). A significance level of P < 0.05 was considered statistically significant.

## Results

### Characteristics of participants

A total of 5,770 participants with HTN were enrolled in this study and divided into two groups: 4,170 in the controlled group and 1,600 in the uncontrolled group (Table 1). The uncontrolled group had a significantly higher percentage of female participants compared to the controlled group (uncontrol; 60.7% vs. control; 53.3%, p < 0.01). Additionally, the mean age of participants in the uncontrolled group was slightly higher than that of the controlled group (uncontrol; 67.1 ± 10.7 vs. control; 66.3 ± 10.3 years, p = 0.01). The uncontrolled group demonstrated a significantly lower eGFR compared to the controlled group (uncontrol; 80.7 ± 20.6 vs. control; 82.1 ± 19.9 mL/min/1.73 m^2^, p = 0.02). Furthermore, the prevalence of proteinuria was significantly higher in the uncontrolled group than in the controlled group (uncontrol; 18.1% vs. control; 14.3%, p < 0.01). The uncontrolled group also reported higher medication compliance (uncontrol; 3.4% vs. control; 6.8%, p = 0.03) and alcohol consumption (uncontrol; 25.1% vs. control; 12.8%, p = 0.02). Although sodium and potassium intakes were significantly lower in the uncontrolled group (sodium: 2952.8 ± 1954.5 vs. 3107.1 ± 1954.9 mmol/L, p = 0.01; potassium: 2523.2 ± 1275.7 vs. 2728.9 ± 1327.8 mmol/L, p < 0.01), the urine Na/K was significantly higher in the uncontrolled group compared to that in the control group (uncontrol; 3.1 vs. control; 2.7, p < 0.01). Patients with CKD in stages 4 and 5 had a significantly higher urine Na/K compared to those in stage 3 (S1 Fig).

**Table 1:** Baseline characteristics of population.

Study participants (n = 5,770)	Control (n = 4,170)	Uncontrol (n = 1,600)	p value
**Sex, n(%)**			<0.01
Male	1,947 (49.7%)	629 (39.3%)	
Female	2,223 (53.3%)	971 (60.7%)	
**Age (years)**	66.3 ± 10.3	67.1 ± 10.7	0.01
**Blood analysis**			
Hemoglobin (g/dL)	13.8 ± 1.5	13.7 ± 1.5	0.79
Glucose (mg/dL)	112.0 ± 30.0	110.5 ± 27.0	0.08
Total cholesterol (mg/dL)	176.1 ± 38.4	182.7 ± 39.5	<0.01
HDL cholesterol (mg/dL)	48.3 ± 11.8	48.9 ± 11.9	0.08
Triglyceride (mg/dL)	139.8 ± 105.6	154.2 ± 112.2	<0.01
eGFR (ml/min/1.73m^2^)	82.1 ± 19.9	80.7 ± 20.6	0.02
**Urine analysis**			
Proteinuria^a^, n(%)	597 (14.3%)	290 (18.1%)	<0.01
Sodium to potassium ratio	2.7 ± 1.7	3.1 ± 1.9	<0.01
**Body measurements**			
Systolic blood pressure (mmHg)	122.5 ± 10.2	147.9 ± 12.2	<0.01
Diastolic blood pressure (mmHg)	73.0 ± 8.6	82.1 ± 11.0	<0.01
Body mass index (kg/m^2^)	25.3 ± 3.4	25.3 ± 3.5	0.92
Waist circumflex (cm)	88.5 ± 9.1	88.3 ± 9.4	0.38
**Health interview, n(%)**			
Poor adherence^b^	100 (6.8%)	55 (3.4%)	0.03
Current smoker	1,569 (37.6%)	527 (32.9%)	0.01
Alcohol drinking	532 (12.8%)	157 (25.1%)	0.02
Regular exercise	1,416 (34.0%)	511 (31.9%)	0.14
**Underlying diseases, n(%)**			
Diabetes mellitus	1,384 (33.2%)	498 (31.1%)	0.10
Dyslipidemia	2,736 (65.6%)	1,047 (65.4%)	0.90
Metabolic syndrome	3,057 (73.3%)	1,208 (75.5%)	0.09
Cardiovascular disease^c^	461 (11.1%)	199 (12.4%)	0.14
**Diet**			
Sodium intake (mg/day)	3107.1 ± 1954.9	2952.8 ± 1954.5	0.01
Potassium intake (mg/day)	2728.9 ± 1327.8	2523.2 ± 1275.7	<0.01

^a^Protienuria is defined as a dipstick test more than 1 positive

^b^HTN medication compliance

^c^Cardiovascular disease include cerebral infarction and myocardial infarction

P values for continuous variables are calculated using the Student t-test or for categorical variables using the chi-square test.

### Association between urine Na/K and BP control

**Fig 3:**
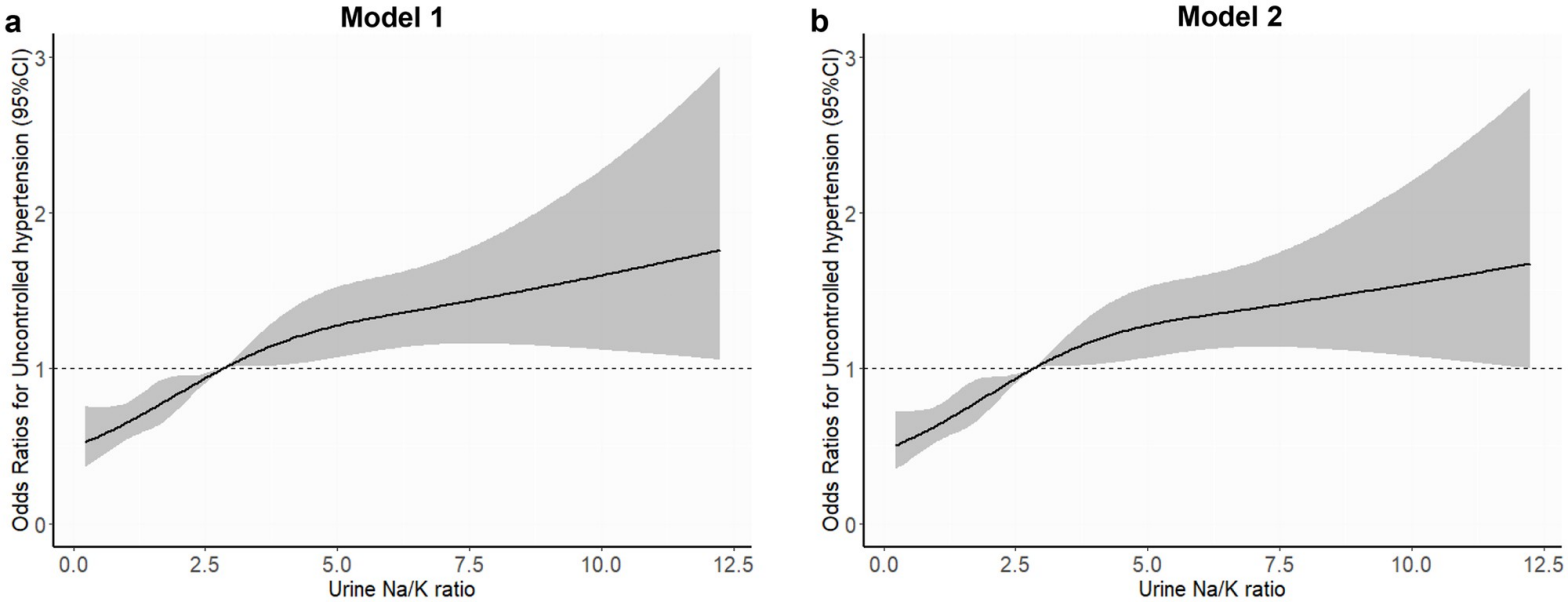
Restricted cubic spline for association between the urine Na/K ratio and uncontrolled hypertension. Model 1 (a) and Model 2 (b). Model 1 was adjusted with sex, age, body mass index, waist circumference, poor adherence, current smoking status, alcohol consumption, regular exercise, diabetes, metabolic syndrome, cardiovascular disease, dyslipidemia, chronic kidney disease, sodium intake, and potassium intake. Model 2 was adjusted with sex, age, body mass index, waist circumference, poor adherence, current smoking status, alcohol consumption, regular exercise, fasting glucose, total cholesterol, HDL cholesterol, triglycerides, estimated glomerular filtration rate (eGFR), sodium intake, and potassium intake.

The participants were categorized into quartiles according to their urine Na/K, sodium intake, and potassium intake. Analysis of urine Na/K quartiles revealed a significant association with BP, with higher quartiles exhibiting significantly higher SBP and MAP than those in the lowest quartile (Fig 2). When stratified by sodium intake, the participants in the highest quartile had significantly lower SBP but higher DBP than those in the lowest quartile (S2 Fig). Similarly, for potassium intake, the third and fourth quartiles showed significantly lower SBP, whereas DBP was significantly higher than in the lowest quartile (S3 Fig). Next, we validated the association between the urine Na/K and uncontrolled HTN using a restricted cubic curve. The restricted cubic curve demonstrated that higher urine Na/K correlated with an increased risk of uncontrolled HTN in both models 1 and 2 (Fig 3). To ensure the stability of our models, we assessed multicollinearity using variance inflation factors (VIF) and found no evidence of multicollinearity (S3 Table).

To explore the correlation between urine Na/K and the risk of uncontrolled HTN, we performed logistic regression analyses. In the crude model, increasing quartiles of the urine Na/K ratio were significantly associated with the risk of uncontrolled HTN (Q1 as a reference; Q2: OR [95% CI] 1.27 [1.07–1.51], p = 0.01; Q3: 1.50 [1.27–1.78], p < 0.01; Q4: 1.85 [1.56–2.20], p < 0.01). Additionally, continuous values of urine Na/K also demonstrated that an increase in the ratio was significantly correlated with the risk of uncontrolled HTN (OR [95% CI]: 1.12 [1.09–1.16], P < 0.01]) (Table 2). Consistent with the crude model, both adjusted models 1 and 2 demonstrated that increasing quartiles and continuous values of urine Na/K were significantly associated with the risk of uncontrolled HTN, as detailed in Table 2. We evaluated the relationship between urine Na/K and both SBP and DBP. Similar to Table 2, the relationship between urine Na/K and SBP also revealed a significant correlation across both quartiles and continuous values (S1 Table). When examining the relationship between urine Na/K and DBP, the highest quartile of the urine Na/K was significantly associated with the elevated DBP compared to the lowest quartile the Crude (OR [95% CI]: 1.98 [1.66–2.36], P < 0.01), Model 1 (OR [95% CI]: 1.44 [1.09–1.91], P = 0.01), and Model 2 (OR [95% CI]: 1.47 [1.11–1.95], P = 0.01). However, the continuous values of urine Na/K were significantly associated with an increased risk of uncontrolled DBP (Crude model: OR [95% CI] 1.08 [1.03–1.13], P < 0.01, Model 1: 1.07 [1.01–1.12], P = 0.02, Model 2: 1.07 [1.01–1.12], P = 0.02) (S2 Table).

**Table 2:** Association between blood pressure control and urine Na/K ratio.

Na/K ratio	Crude model		Adjusted model*			
			Model 1^a^		Model 2^b^	
	OR (95% CI)	p-value	OR (95% CI)	p-value	OR (95% CI)	p-value
**Quartiles of urine Na/K ratio**						
Quartile 1	1 (reference)		1 (reference)		1 (reference)	
Quartile 2	1.27 (1.07–1.51)	0.01	1.26 (1.06–1.49)	0.01	1.28 (1.07–1.52)	0.01
Quartile 3	1.50 (1.27–1.78)	<0.01	1.50 (1.27–1.78)	<0.01	1.55 (1.30–1.83)	<0.01
Quartile 4	1.85 (1.56–2.20)	<0.01	1.83 (1.55–2.17)	<0.01	1.88 (1.59–2.23)	<0.01
**Continuous values of urine Na/K ratio**						
Urine Na/K ratio	1.12 (1.09–1.16)	<0.01	1.13 (1.09–1.16)	<0.01	1.13 (1.09–1.16)	<0.01

^a^Model was adjusted with sex, age, body mass index, waist circumference, poor adherence, current smoking status, alcohol consumption, regular exercise, diabetes, metabolic syndrome, cardiovascular disease, dyslipidemia, chronic kidney disease, sodium intake, and potassium intake.

^b^Model was adjusted with sex, age, body mass index, waist circumference, poor adherence, current smoking status, alcohol consumption, regular exercise, fasting glucose, total cholesterol, HDL cholesterol, triglycerides, estimated glomerular filtration rate (eGFR), sodium intake, and potassium intake.

### Association between urine Na/K and BP control through subgroup analysis

We performed a subgroup analysis to investigate the association between urine Na/K and BP control across different subgroups, including sex, prevalence of proteinuria, and prevalence of CKD. In both male and female, an increase in the urine Na/K was associated with uncontrolled HTN (Table 3). Notably, participants with CKD showed that an increase in urine Na/K was associated with uncontrolled HTN (Crude: OR [95% CI] 1.20 [1.09–1.31], p < 0.01; Model 1: 1.19 [1.09–1.32], p < 0.01; Model 2: 1.19 [1.08–1.31], p < 0.01). Therefore, urine Na/K may be affected by kidney function. Similarly, participants with proteinuria also demonstrated that an increase in urine Na/K was associated with uncontrolled HTN (Crude: OR [95% CI] 1.20 [1.09–1.31], p < 0.01; Model 1: 1.16 [1.05–1.27], p < 0.01; Model 2: 1.16 [1.06–1.28], p < 0.01) (Table 3).

**Table 3:** Subgroup analysis for association between blood pressure control and urine Na/K ratio.

Subgroup	Crude model		Adjusted model*				p for interaction
			Model 1^a^		Model 2^b^		
	OR (95% CI)	p-value	OR (95% CI)	p-value	OR (95% CI)	p-value	
**Sex**							0.526
Male (n = 2,576)	1.12 (1.06–1.17)	<0.01	1.11 (1.06–1.16)	<0.01	1.10 (1.05–1.16)	<0.01	
Female (n = 3,194)	1.14 (1.09–1.19)	<0.01	1.14 (1.09–1.19)	<0.01	1.14 (1.10–1.20)	<0.01	
**Proteinuria**							0.247
Yes (n = 887)	1.20 (1.09–1.31)	<0.01	1.16 (1.05–1.27)	<0.01	1.16 (1.06–1.28)	<0.01	
No (n = 4,883)	1.23 (1.09–1.17)	<0.01	1.14 (1.10–1.18)	<0.01	1.14 (1.10–1.18)	<0.01	
**CKD**							0.174
Yes (n = 704)	1.20 (1.09–1.31)	<0.01	1.19 (1.09–1.32)	<0.01	1.19 (1.08–1.31)	<0.01	
No (n = 5,066)	1.12 (1.08–1.15)	<0.01	1.12 (1.08–1.16)	<0.001	1.12 (1.08–1.16)	<0.01	

^a^Model was adjusted with sex, age, body mass index, waist circumference, poor adherence, current smoking status, alcohol consumption, regular exercise, diabetes, metabolic syndrome, cardiovascular disease, dyslipidemia, chronic kidney disease, sodium intake, and potassium intake, except subgroup variable.

^b^Model was adjusted with sex, age, body mass index, waist circumference, poor adherence, current smoking status, alcohol consumption, regular exercise, fasting glucose, total cholesterol, HDL cholesterol, triglycerides, estimated glomerular filtration rate (eGFR), sodium intake, and potassium intake, except subgroup variable.

## Discussion

In this study, we investigated the association between urine Na/K and uncontrolled HTN. Our results demonstrated that a higher urine Na/K was significantly associated with uncontrolled BP, even after adjusting for various confounding factors (Table 2). Participants were excluded based on age, missing data, cancer, and pregnancy (Fig 1). While these exclusions may introduce selection bias, potentially limiting the generalizability of our findings, they were necessary to reduce confounding factors. Pregnancy induces significant changes in hemodynamics and cardiac output, directly affecting BP [30, 31]. Cancer and hypertension share overlapping pathophysiological mechanisms, such as inflammation and oxidative stress, and cancer treatments can both directly cause hypertension and indirectly impact BP through nephrotoxicity [32, 33]. Additionally, BP levels are generally lower in children and adolescents compared to adults, which further justifies the exclusion of younger participants [34].

To assess normality, histogram and Q-Q plots were employed, as shown in S4 and S5 Figs. In addition, skewness and kurtosis are close to 0, indicating a normal distribution (S4 Table). Specifically, while the SBP values did not fully conform to normality based on Q-Q plots (S4 Fig), our study sample size (n = 5,770) is sufficiently large to minimize the impact of the violation of normality assumptions. According to the central limit theorem, with large samples, the sampling distribution tends to approximate normality regardless of the shape of the underlying data [35].

Guidelines for target BP in patients with HTN are tailored according to individual risk factors, including CVD risk and age [8, 9]. In this study, we defined uncontrolled HTN as an SBP ≥ 140 mmHg or a DBP ≥ 90 mmHg. This definition is in line with the thresholds established by the Korean Society of Hypertension [36], which is particularly relevant given that our study population consisted of participants from the KNHANES, representing a broad cross-section of the general Korean population.

The World Health Organization recommends consuming less than 2,000 mg of sodium and at least 3.500 mg of potassium daily to maintain healthy blood pressure levels, emphasizing that reducing sodium intake can be an effective strategy for lowering blood pressure [37]. The average daily sodium intake in the general Korean population was approximately 3,477.2 to 3,889.6 mg [38], and an average daily potassium intake was around 2,934.7 to 3,232.0 mg [39]. In comparison, participants in this study had an average sodium intake of 3,115 mg and an average potassium intake of 2,720 mg. This indicates that the study population had lower sodium and potassium intakes compared to the general Korean population. Although the amounts of sodium and potassium intake affect BP control, the role of the urine Na/K, which reflects the balance of these electrolytes in the diet and their regulation by the kidneys, is unclear. Notably, the uncontrolled HTN group not only had reduced sodium and potassium intakes but also included a higher proportion of females compared to the control group (uncontrolled: n = 971 (60.7%) vs. control: n = 2,223 (53.3%), Table 1). When participants were divided into quartiles based on urine Na/K and sodium and potassium intake, the quartile with the lowest urine Na/K exhibited lower SBP and MAP (Fig 2). However, the quartile with the lowest sodium intake had a higher SBP than that with the highest sodium intake (S2 Fig). Additionally, the quartile with the highest potassium intake displayed a lower SBP but higher DBP (S3 Fig). These results suggest that the relationship between the sodium and potassium excreted by the kidneys, rather than the absolute intake of these electrolytes, may be more critical for BP control. Previously, urine Na/K was positively associated with uncontrolled HTN in patients with CKD [40]. Consistent with the findings of a previous study, our results showed that a higher urine Na/K was significantly associated with uncontrolled HTN in patients with CKD (Table 3). Interestingly, the uncontrolled group exhibited higher medication adherence compliance compared to the controlled group (Table 1), suggesting that individuals with elevated urine Na/K may require more comprehensive BP management strategies, such as exercise, optimized antihypertensive drug regimens, and dietary modifications, to achieve optimal BP control. In this study, we performed multi-variable logistic regression analyses using two models. In Model 1, we included categorical variables such as CKD, DM, and dyslipidemia to account for the presence of these conditions. Model 2 utilized continuous variables like fasting glucose and eGFR. Interestingly, the adjusted OR in both models were similar to the crude OR (Table 2), suggesting that the confounding factors had minimal impact on the association between urine Na/K and uncontrolled HTN. These results further reinforce that urine Na/K is independently associated with uncontrolled HTN.

This study had some limitations. First, as a cross-sectional study, we could not establish a causal relationship between urine Na/K and uncontrolled HTN. Second, sodium and potassium intake were determined by 24-h dietary recall, which may not accurately reflect the actual intake. However, previous studies have shown that the 24-h dietary recall method used in the KNHANES is a reliable tool for assessing food and nutrient intake [41, 42]. Third, the relationship between BP and urine Na/K was assessed using single measurements of BP and spot urine samples, which may not be as precise as 24-h ambulatory BP monitoring and 24-h urine collection.

Despite several limitations, this study has several strengths. First, we used a large nationally representative sample from the KNHANES to enhance the generalizability of the findings, allowing for broader interpretations and applications in public health strategies. Second, eGFR was calculated using the MDRD formula. While the MDRD formula is widely used, it tends to underestimate GFR at higher levels (above 60 mL/min/1.73 m^2^) and may not be as accurate in certain populations, such as the elderly, individuals with extremes of body size or muscle mass, and those on vegetarian diets [43, 44]. Third, although spot urine samples were used, the urine Na/K is a practical and easily assessed measure that can be used to screen and monitor patients, making it a valuable tool for managing HTN in typical clinical environments. Moreover, BP measurements in the KNHANES are a good reflection of actual BP readings. Although spot urine samples offer a convenient and non-invasive method for assessing the Na/K ratio, they may not fully capture daily variations compared to 24-hour urine collections. Nevertheless, because the measurement is expressed as a ratio rather than absolute values of Na and K, it compensates for differences in diurnal excretion and is considered an effective indicator. Further studies are needed to establish a causal relationship between urine Na/K and uncontrolled HTN. Future research should employ multiple 24-h urine collections and ambulatory blood pressure monitoring to provide more precise assessments of the relationship between urine Na/K and BP control over time.

In conclusion, this study identified that an elevated urine Na/K is independently associated with uncontrolled HTN in the Korean population and in patients with CKD. Even when office BP appears well-controlled, an elevated urine Na/K may help identify patients at risk for uncontrolled HTN at home or through ambulatory BP monitoring. Our findings suggest that monitoring urine Na/K could serve as an effective tool for identifying individuals at risk of uncontrolled HTN.

## Supporting information

S1 Fig Comparison of the urine Na/K between CKD stage 3 and CKD stage 4–5. The box plot illustrates the distribution of the urine Na/K in individuals with CKD stage 3 and CKD stage 4–5. Each boxplot illustrates the median (central line), the interquartile range (box edges), and the range (whiskers), for each quartile. P values were calculated using Student t-test *p< 0.05.
(TIF)

S2 Fig Boxplots of blood pressure metrics across sodium intake quartiles. The boxplots representing the distribution of systolic blood pressure (SBP, a), diastolic blood pressure (DBP, b) and mean arterial pressure (MAP, c) across different quartiles of the sodium intake. Each boxplot illustrates the median (central line), the interquartile range (box edges), and the range (whiskers), for each quartile. P values were calculated using one-way ANOVA with Tukey’s post-hoc analysis; ****p< 0.0001 and ns = not significant.
(TIF)

S3 Fig Boxplots of blood pressure metrics across potassium intake quartiles. The boxplots representing the distribution of systolic blood pressure (SBP, a), diastolic blood pressure (DBP, b) and mean arterial pressure (MAP, c) across different quartiles of the potassium intake. Each boxplot illustrates the median (central line), the interquartile range (box edges), and the range (whiskers), for each quartile. P values were calculated using one-way ANOVA with Tukey’s post-hoc analysis; *p< 0.05, ***p< 0.001, ****p< 0.0001 and ns = not significant.
(TIF)

S4 Fig Histogram and Q-Q plot of SBP. (a) Histogram of SBP showing the distribution of values. (b) Q-Q plot of SBP residuals comparing observed values to a normal distribution. Abbreviation: SBP, systolic blood pressure.
(TIF)

S5 Fig Histogram and Q-Q plot of DBP. (a) Histogram of DBP showing the distribution of values. (b) Q-Q plot of DBP residuals comparing observed values to a normal distribution. Abbreviation: DBP, diastolic blood pressure.
(TIF)

S1 Table Association between systolic blood pressure control and urine Na/K ratio. (DOCX)

S2 Table Association between diastolic blood pressure control and urine Na/K ratio. (DOCX)

S3 Table Variance inflation factors of each confounding factors. (DOCX)

S4 Table Descriptives. (DOCX)
