# Supplementary figures and images for: Association between blood pressure control in hypertension and urine sodium to potassium ratio: From the Korea National Health and Nutrition Examination Survey (2016–2021)
Source: PLoS One. 2024 Nov 26;19(11):e0314531. doi: 10.1371/journal.pone.0314531 (PMC11594522; doi:10.1371/journal.pone.0314531)

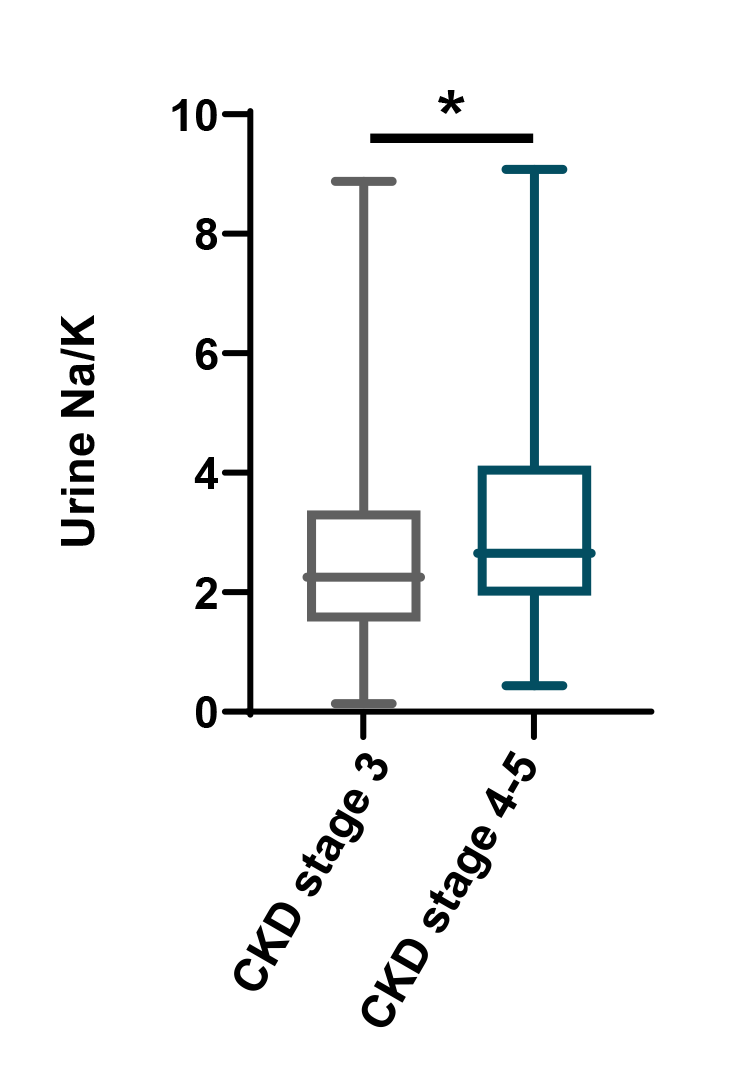

Supplement: S1 Fig — The box plot illustrates the distribution of the urine Na/K in individuals with CKD stage 3 and CKD stage 4–5. Each boxplot illustrates the median (central line), the interquartile range (box edges), and the range (whiskers), for each quartile. P values were calculated using Student t-test *p< 0.05. (TIF) [file pone.0314531.s001.tif]

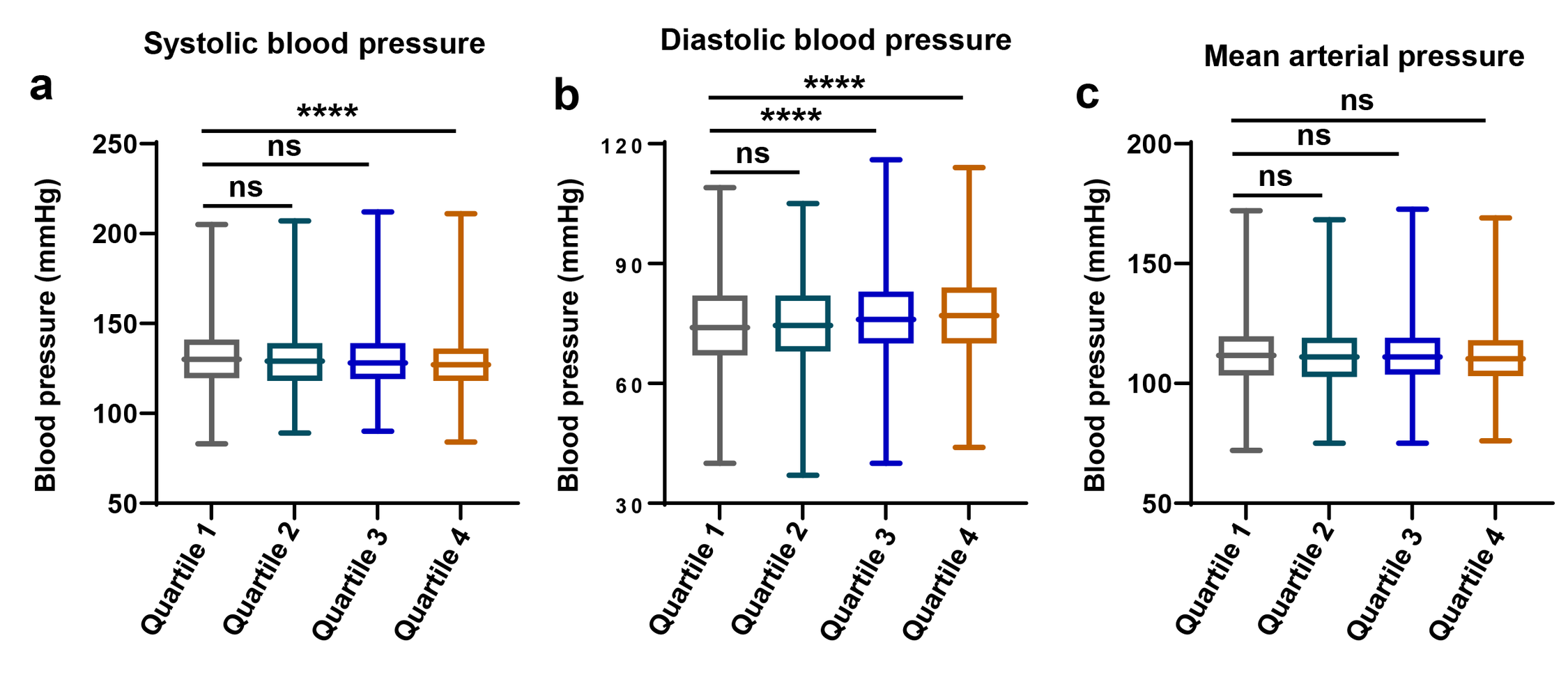

Supplement: S2 Fig — The boxplots representing the distribution of systolic blood pressure (SBP, a), diastolic blood pressure (DBP, b) and mean arterial pressure (MAP, c) across different quartiles of the sodium intake. Each boxplot illustrates the median (central line), the interquartile range (box edges), and the range (whiskers), for each quartile. P values were calculated using one-way ANOVA with Tukey’s post-hoc analysis; ****p< 0.0001 and ns = not significant. (TIF) [file pone.0314531.s002.tif]

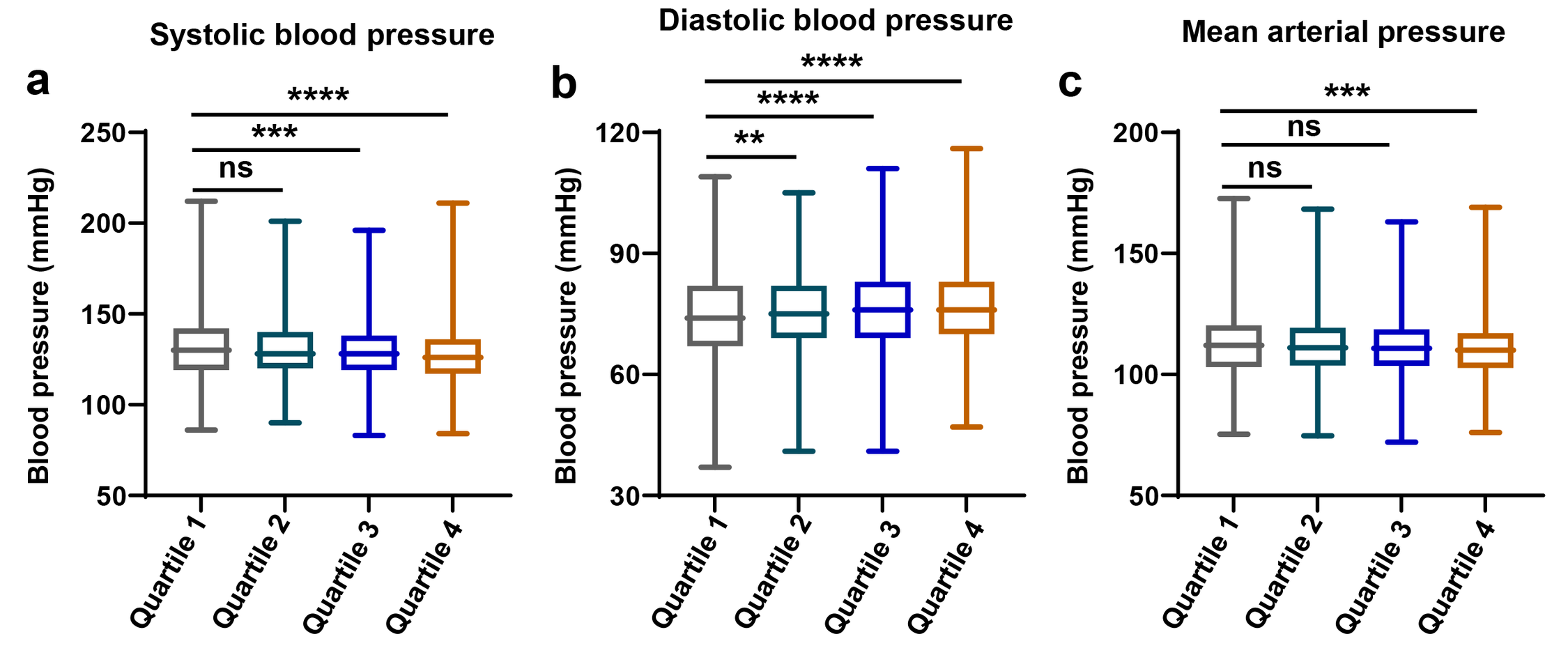

Supplement: S3 Fig — The boxplots representing the distribution of systolic blood pressure (SBP, a), diastolic blood pressure (DBP, b) and mean arterial pressure (MAP, c) across different quartiles of the potassium intake. Each boxplot illustrates the median (central line), the interquartile range (box edges), and the range (whiskers), for each quartile. P values were calculated using one-way ANOVA with Tukey’s post-hoc analysis; *p< 0.05, ***p< 0.001, ****p< 0.0001 and ns = not significant. (TIF) [file pone.0314531.s003.tif]

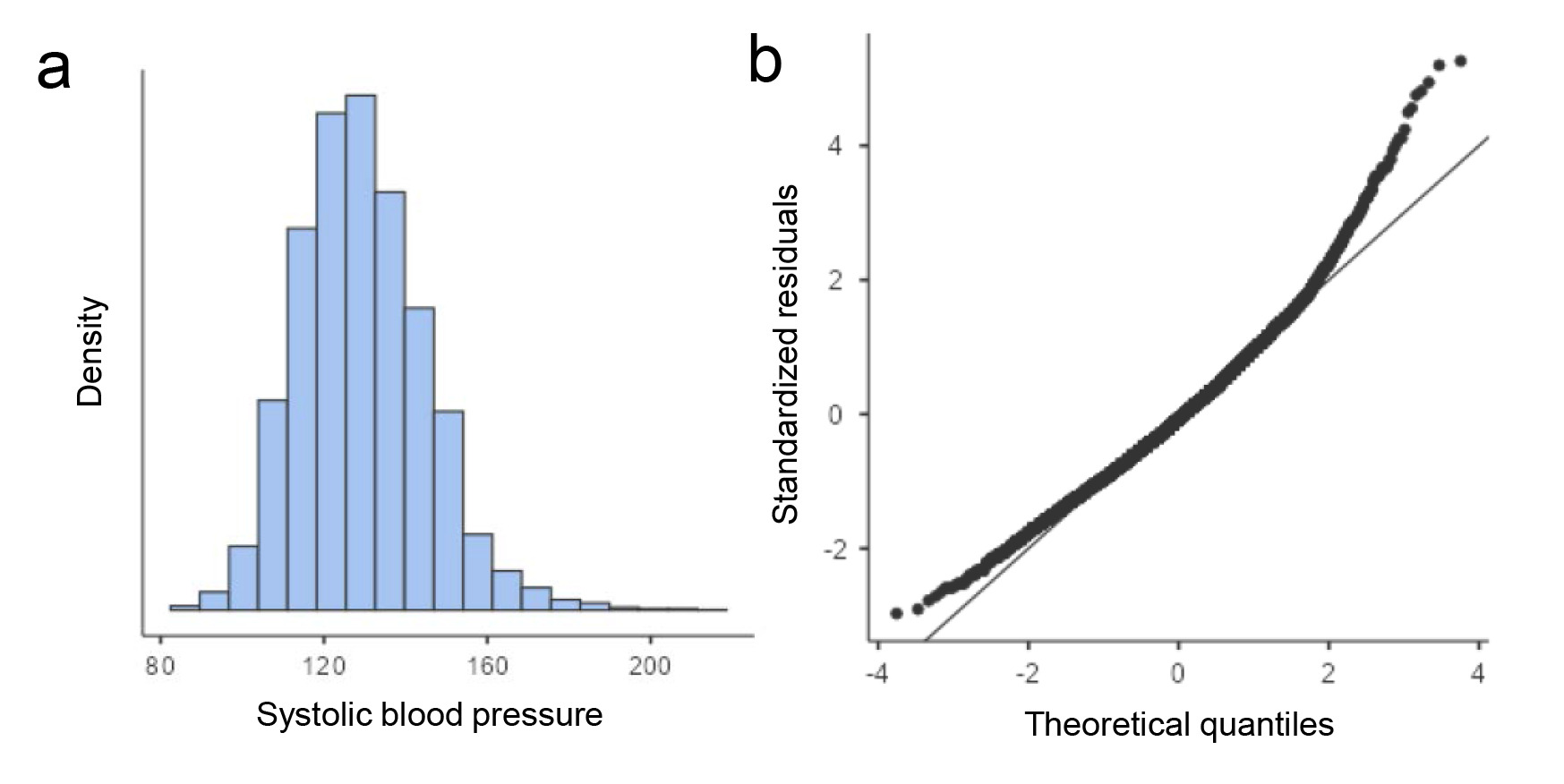

Supplement: S4 Fig — (a) Histogram of SBP showing the distribution of values. (b) Q-Q plot of SBP residuals comparing observed values to a normal distribution. Abbreviation: SBP, systolic blood pressure. (TIF) [file pone.0314531.s004.tif]

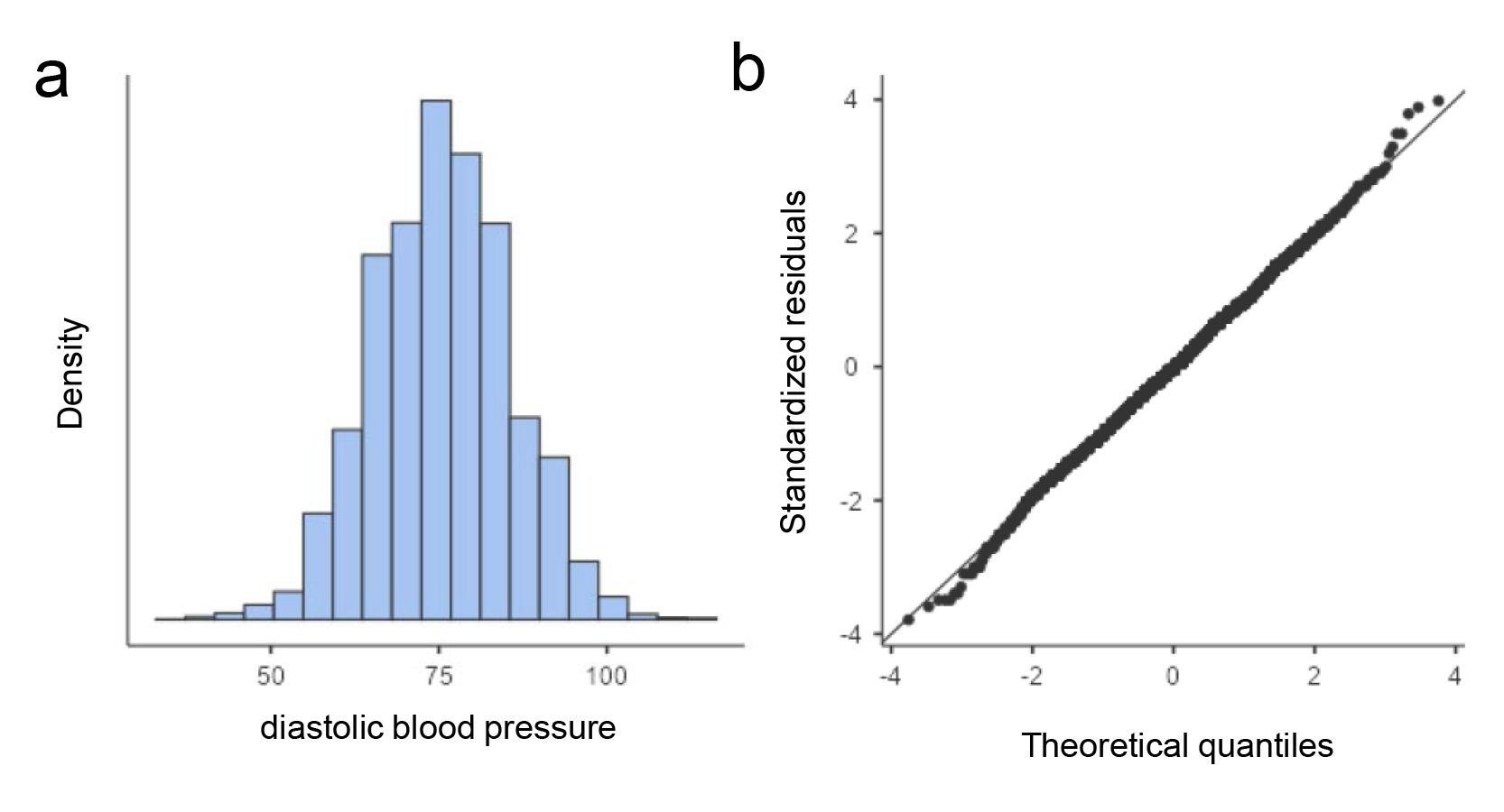

Supplement: S5 Fig — (a) Histogram of DBP showing the distribution of values. (b) Q-Q plot of DBP residuals comparing observed values to a normal distribution. Abbreviation: DBP, diastolic blood pressure. (TIF) [file pone.0314531.s005.tif]
