# Supplementary material for: Association between blood pressure control in hypertension and urine sodium to potassium ratio: From the Korea National Health and Nutrition Examination Survey (2016–2021)
Source: PLoS One. 2024 Nov 26;19(11):e0314531. doi: 10.1371/journal.pone.0314531 (PMC11594522; doi:10.1371/journal.pone.0314531)
Supplement: S1 Table — (DOCX) [file pone.0314531.s006.docx]

**S1 Table. Association between systolic blood pressure control and urine Na/K ratio**

| **Na/K ratio** | **Crude model** | | **Adjusted model*** | | | |
| --- | --- | --- | --- | --- | --- | --- |
|  |  |  | **Model 1**^a^ | | **Model 2**^b^ | |
|  | **OR (95% CI)** | **p-value** | **OR (95% CI)** | **p-value** | **OR (95% CI)** | **p-value** |
| **Quartiles of urine Na/K ratio** |  |  |  |  |  |  |
| Quartile 1 | 1 (reference) |  | 1 (reference) |  | 1 (reference) |  |
| Quartile 2 | 1.37  (1.14 – 1.65) | 0.01 | 1.35  (1.12 – 1.63) | 0.02 | 1.37  (1.13 – 1.65) | 0.01 |
| Quartile 3 | 1.57  (1.31 – 1.87) | <0.01 | 1.60  (1.33 – 1.92) | <0.01 | 1.64  (1.36 – 1.97) | <0.01 |
| Quartile 4 | 1.98  (1.66 – 2.36) | <0.01 | 2.01  (1.68 – 2.41) | <0.01 | 2.05  (1.71 – 2.46) | <0.01 |
| **Continuous values of urine Na/K ratio** |  |  |  |  |  |  |
| Urine Na/K ratio | 1.13  (1.09 – 1.16) | <0.01 | 1.14  (1.10 – 1.18) | <0.01 | 1.14  (1.10 – 1.18) | <0.01 |

^a^Model was adjusted with sex, age, body mass index, waist circumference, poor adherence, current smoking status, alcohol consumption, regular exercise, diabetes, metabolic syndrome, cardiovascular disease, dyslipidemia, chronic kidney disease, sodium intake, and potassium intake.

^b^Model was adjusted with sex, age, body mass index, waist circumference, poor adherence, current smoking status, alcohol consumption, regular exercise, fasting glucose, total cholesterol, HDL cholesterol, triglycerides, estimated glomerular filtration rate (eGFR), sodium intake, and potassium intake.
