# Supplementary material for: Association between blood pressure control in hypertension and urine sodium to potassium ratio: From the Korea National Health and Nutrition Examination Survey (2016–2021)
Source: PLoS One. 2024 Nov 26;19(11):e0314531. doi: 10.1371/journal.pone.0314531 (PMC11594522; doi:10.1371/journal.pone.0314531)
Supplement: S3 Table — (DOCX) [file pone.0314531.s008.docx]

**S3 Table. Variance inflation factors of each confounding factors**

| **Model 1** | | **Model 2** | |
| --- | --- | --- | --- |
| **Factor** | **VIF** | **Factor** | **VIF** |
| Sex | 1.76 | Sex | 1.80 |
| Age | 1.34 | Age | 1.40 |
| Body mass index | 4.27 | Body mass index | 4.28 |
| Waist circumference | 4.34 | Waist circumference | 4.41 |
| Poor adherence | 1.01 | Poor adherence | 1.01 |
| Current smoking status | 1.16 | Current smoking status | 1.17 |
| Alcohol consumption | 1.34 | Alcohol consumption | 1.40 |
| Regular exercise | 1.05 | Regular exercise | 1.05 |
| Diabetes | 1.12 | Fasting glucose | 1.08 |
| Metabolic syndrome | 1.72 | Total cholesterol | 1.25 |
| Cardiovascular disease | 1.04 | HDL cholesterol | 1.42 |
| Dyslipidemia | 1.32 | Triglycerides | 1.37 |
| Chronic kidney disease | 1.07 | eGFR | 1.14 |
| Sodium intake | 1.55 | Sodium intake | 1.55 |
| Potassium intake | 1.48 | Potassium intake | 1.49 |
