# Supplementary material for: Association between blood pressure control in hypertension and urine sodium to potassium ratio: From the Korea National Health and Nutrition Examination Survey (2016–2021)
Source: PLoS One. 2024 Nov 26;19(11):e0314531. doi: 10.1371/journal.pone.0314531 (PMC11594522; doi:10.1371/journal.pone.0314531)
Supplement: S4 Table — (DOCX) [file pone.0314531.s009.docx]

**S4 Table. Descriptives**

| **Descriptives** | **SBP** | **DBP** |
| --- | --- | --- |
| **N** | 5770 | 5770 |
| **Mean** | 130 | 75.5 |
| **Median** | 128 | 75.0 |
| **Standard deviation** | 15.7 | 10.2 |
| **Minimum** | 83.0 | 37.0 |
| **Maximum** | 212 | 116 |
| **Skewness** | 0.599 | 9.62e-4 |
| **Kurtosis** | 1.14 | 0.128 |

Abbreviation: SBP; systolic blood pressure, DBP; diastolic blood pressure, urine Na/K; urine sodium to potassium ratio.
